# Liquid Chromatography-Tandem Mass Spectrometry Detection of Human and Veterinary Drugs and Pesticides in Surface Water

**DOI:** 10.1155/2023/6350669

**Published:** 2023-10-16

**Authors:** Xiang-Pu Zhang, Shu Zhang, Chun-Yan Xu, Wei-Wei Li, Hai-Bo Ling, Yang Luo, Kang Jian, Tao Li, Chuan Yi

**Affiliations:** ^1^National Key Laboratory of Environmental Health Risk Assessment for Environmental Protection, Hubei Provincial Academy of Eco-Environmental Sciences, Wuhan 430070, China; ^2^Wuhan Ecological Environmental Monitoring Center, Department of Ecology and Environment of Hubei Province, Wuhan 430070, China

## Abstract

Antibiotics and pesticides are widespread in most rivers and lakes due to the overuse of antibiotics and pesticides, but there are few methods for simultaneous analysis of antibiotics and pesticides in aquatic environments. To address this knowledge gap, a concise and sensitive analytical method is proposed in which three classes of human and veterinary drugs (sulfonamides, macrolides, and hormones) and two classes of pesticides (organophosphorus and neonicotinoids) are simultaneously extracted and determined in surface water. The solid-phase extraction column with Cleanert PEP-2 was preconditioned sequentially with 6 mL of methanol, ultrapure water, and citric acid buffer (pH 3.0) each for simultaneous extraction and further purification. The forty-seven target analytes were analysed by LC-MS/MS in positive and negative ion modes. The LC separation was performed using a Sigma-Aldrich C_18_ column with 0.1% formic acid in water and acetonitrile as a gradient eluting mobile phase in positive ion mode. The internal standard method was used to overcome the inevitable matrix effects in LC-MS/MS analysis. The matrix effects of most target analytes were in the range of 27–151%. The recoveries of forty analytes in the three concentrations (10, 50, and 100 ng L^−1^) of surface water spiked samples ranged from 41 to 127%. The method quantitative limits of the analytes were in the range of 0.40–5.49 ng L^−1^. Application of the method to analyze samples in the eight runoff outlets of the Pearl River Delta showed that some antibiotics and pesticides were detected, and the concentration of parathion was as high as 154 ng L^−1^. A powerful tool for quickly and efficiently screening for contaminants in surface water has been presented.

## 1. Introduction

Human and veterinary drugs (HVDs), considered emerging pollutants, are attracting increased attention worldwide [1, 2]. They have been increasingly administered to prevent and treat microbial infections in humans and animals [3–5] and are commonly added to animal feeds as supplements to promote the growth of animals in livestock and aquaculture [6–8]. Upon administration, fractions of pharmaceuticals can enter into different environmental compartments in their parent forms or as metabolites through excretion of urine and feces [9]. These chemical residues can be further discharged into aquatic environments through the direct disposal of human and animal wastes [10, 11] or effluents from municipal sewage treatment plants [12, 13]. HVDs are not easy to combine with solid materials. Parent drugs and their metabolites are often hydrophilic and easily reach the aquatic environment, posing a threat to surface and ground water. Consequently, HVDs have been detected in surface water [14] and groundwater [15] in bioactive and bioavailable forms, leading to undesirable effects on nontarget organisms and drinking water supplies, as well as contamination of food [16]. For example, HVDs have been detected in a number of river systems in China [17–26], especially sulfonamides (SAs) and macrolides (MLs) which have been detected frequently, indicating that bacterial resistance and potential ecological risks caused by HVDs in water should not be overlooked.

Organophosphates (OPs) and neonicotinoids (NNIs) have been used to protect crops against pests as they exhibit a broad spectrum of insecticidal activities and low toxicity to mammals [27]. The use of organophosphorus pesticides and neonicotinoid pesticides accounted for one-third of the global pesticide market share. Although some OPs have been banned and replaced by NNIs, pollution of the aquatic environment has become a serious problem due to the toxicity and abuse of OPs, causing a risk to human health [28]. NNIs may have potential ecotoxic effects on pollinators such as bees and invertebrates [29]. The European Union banned the use of NNIs on flowering crops that attract bees in 2013 [30]. However, NNIs remain widely used around the world due to their low price and significant effects. These pesticides may remain in agricultural products consumed by humans and enter into the digestive tract. They can also be washed off plant surfaces by rain and discharged into river systems. Numerous methods have been developed to detect OPs and NNIs in fruits [31, 32], vegetables [33, 34], and honey [35, 36].

Despite the availability of numerous methods for separate detection of HVDs and pesticide residues in a variety of sample matrices [31, 32, 36–39], relatively fewer methods are available for simultaneously purifying and measuring SAs, MLs, hormones (HMs), OPs, and NNIs in environmental samples. Previous studies on pretreatment and instrumental analysis of surface water samples have targeted one group or a narrow range of compounds only [39, 40]. For combined extraction, clean-up, and analytical separation, there seems to be a considerable challenge due to the amphoteric nature, the wide range of chemical families, and the wide polarity range of pollutants present.

To address this issue, the present study is set out to develop a rapid, sensitive, and validated method for simultaneously extracting, purifying, and detecting 47 analytes extracted from surface water. Target analytes include 47 human and veterinary drugs and pesticide residues (Supplemental Data (SD); Table S1), which are commonly used chemicals in aquaculture and agriculture. As a worldwide population and agricultural country, the dramatic increase in the use of HVDs and pesticides has led to the frequent detection of these chemicals in most rivers in China [17–27]. Therefore, it is very urgent and necessary to provide an analytical method for rapid screening of multiple pollutants. Considering that simultaneous detection of multiple analytes may cause mutual interference, the instrument methods were first optimized, and the analytes were effectively separated via liquid chromatography. For sample pretreatment, a solid-phase extraction method was selected and optimized. It was verified by a matrix-spiked experiment and successfully applied to environmental water samples. The results showed that this pretreatment method has acceptable and stable recoveries.

## 2. Materials and Methods

### 2.1. Materials and Chemicals

Standards of SAs and OPs were obtained from O2si Smart Solutions (Charleston, SC, USA). Other MLs and HMs were purchased from ChemTek (Worcester, MA, USA). The ten NNIs compounds were obtained from Manhage Bio-Tech (Beijing, China) (Supplemental Data; Table S1). Dinotefuran, imidaclothiz, and isotope-labeled standards (sulfachloropyridazine-*d*_3_ and clothianidin-*d*_3_) were purchased from Dr. Ehrenstorfer (Augsburg, Germany). Isotope-labeled standards imidaclothiz-*d*_4_ and acetamiprid-*d*_3_ were obtained from C/D/N isotopes (Quebec, Canada), while estradiol-*d*_2_ and diethylstilbrstrol-*d*_8_ were obtained from Manhage Bio-Tech (Beijing, China). Another three isotope-labeled standards tilmicosin-*d*_3_, sulfamethoxzole-*d*_4_, and sulfamethazine-*d*_4_ were obtained from Toronto Research Chemicals (North York, ON, Canada). All standards of the target analytes are solutions (concentration of 100 mg/L), and the isotope-labeled standards are solid. The solid standards were weighed with an electronic balance (Ohaus Instruments, Changzhou, China) with an accuracy of 0.00001 g and dissolved in methanol (concentration of 100 mg/L) prior to use.

Formic acid (LCMS grade) and disodium ethylenediamine tetraacetate (Na_2_EDTA, LC grade) were purchased from Sigma-Aldrich (St. Louis, MO, USA). Citric acid monohydrate and trisodium citrate dihydrate were purchased from Chemical Reagent Factory (Guangzhou, China). Ultrapure water was provided by a MilliQ water purification system (Millipore, Billerica, Germany). Methanol and acetonitrile of HPLC and LCMS grades were purchased from Oceanpak (Gothenburg, Sweden) and Thermo Fisher Scientific (Waltham, MA, USA), respectively. The Cleanert PEP-2 cartridges (6 mL, 200 mg) were supplied by Agela Technologies (Tianjin, China). Glass fiber filters (pore size 0.7 *μ*m) were purchased from Whatman International (Maidstone, England) and heated at 450°C for 4 h in order to pyrolyze any organic material present.

### 2.2. Optimization of Sample Pretreatment

Solid-phase extraction cartridges were selected for extraction and purification. The pH value of water samples and the amount of Na_2_EDTA used were significant to the recovery of antibiotics [39]. These two parameters were optimized as part of method development efforts. The separation efficacy of a liquid chromatography column is directly related to its diameter and length, the particle size of the packing materials, additives, mobile phase gradients, and flow rates. These parameters were thus rigorously examined with the analytes selected.

Water samples were filtered through 0.7 *μ*m glass fiber filters. One liter of filtered water was acidified to pH 3.0 with 1.0 mol L^−1^ H_2_SO_4_, followed by the addition of 0.2 g Na_2_EDTA. Surrogate standards (sulfamethazine-*d*4, sulfamethoxypyridazine-*d*3, acetamiprid-*d*3, clothianidin-*d*3, and diethylstilbrstrol-*d*8 at 1 *μ*g mL^−1^; 20 *μ*L) were added to each sample prior to extraction. A Cleanert PEP-2 cartridge (6 mL, 200 mg) was preconditioned sequentially with 6 mL of methanol, ultrapure water, and citric acid buffer (pH 3.0) each. Thereafter, the samples were passed through the cartridge at a flow rate of 3.0 mL min^−1^. The cartridge was then rinsed with 6 mL of ultrapure water and dried under N_2_ gas for 1 h. Each dried cartridge was eluted with 6 mL of methanol. Target analytes were collected in a 10 mL glass vial, volume-reduced under N_2_ purge to approximately 10 *μ*L, and dissolved in a mixture of methanol : water (1 : 9 in volume) containing 0.1% formic acid to a final volume of 1 mL. Suspended particles were removed by 0.22 *μ*m membrane filtration. Internal standards (sulfamethoxazole-*d*_4_, tilmicosin-*d*_3_, imidacloprid-*d*_4_, and estradiol-*d*_2_ at 1 *μ*g mL^−1^; 20 *μ*L) were added to each extract which was stored at −18°C until LC-MS/MS analysis. The retention time of the internal standard is close to that of the analyte and evenly distributed.

Matrix-spiked samples were prepared from water (collected from the park of Qi XingGang, Guangdong province) spiked with all analytes at three concentrations (10, 50, and 100 ng L^−1^), and each recovery test was conducted in triplicate. These three concentrations were comparable to those in the environment and also provided an appropriate concentration range for examining the robustness of the method.

### 2.3. Instrumental Analysis

All analytes were measured with a triple quadrupole 5500 electrospray ionization-tandem mass spectrometer (AB SCIEX; Redwood City, CA) equipped with an LC-20A high-performance liquid chromatograph (Shimadzu, Japan) in the multiple-reaction monitoring mode. Ion source gas, curtain gas, collision gas, temperature, and ionspray voltage were optimized by flow injection analysis tuning. The detailed parameters are shown in Table 1. The mass spectrometer parameters were optimized for each analyte by direct infusion of each standard solution in methanol at a concentration of 100 ng mL^−1^. The precursor and product ions, declustering potential, collision energy, entrance potential, and collision cell exit potential were optimized and confirmed to have the best instrument response through the liquid chromatography-mass spectrometry system (Tables 2 and 3). Two ion fragments were selected for qualitative and quantitative determination of each analyte to obtain high selectivity and sensitivity. The choice of positive and negative ion modes was based mainly on the physicochemical properties and instrumental responses of the analytes. The majority were analysed in positive ionisation mode, with the exception of the four steroids listed (diethylstilbestrol, estradiol, estrone, and estriol), which were analysed in negative ionisation mode. All analytes (except for diethylstilbestrol, estradiol, estrone, and estriol) were detected in the positive ion mode. The selected precursor ions for all the analytes in the positive mode were [M+H]^+^ while those in the negative mode were [M−H]^−^.

### 2.4. Site Description and Sample Collection

As one of the most economically developed regions in China, the Pearl River Delta (PRD) has experienced explosive economic growth over the past 30 years, leading to rapid industrialization and urbanization and resulting in a large population increase [41]. The living and consumption levels of residents have been greatly improved, which has brought about non-negligible impacts on the ecological environment of the region. The Pearl River Basin is a complex river basin composed of numerous main streams and tributaries, which are greatly affected by the surrounding human activities [42]. The eight major runoff outlets are imported into the South China Sea. Water samples were collected from the eight major runoff outlets at the PRD, located in Guangdong Province, Southern China, on August 11, 2018. The collected samples are odorless and contain a small amount of particles. The position of the sampling sites has been given by the previous study [41] (Supplemental Data; Table S2). The collected water samples were pretreated with a filter membrane within 48 hours. The water samples used for matrix spikes come from the park of Qi Xing Gang, which is located in Guangdong Province and has a target-free environmental pollution in the landscape protection zone. The water samples are odorless and free from interference.

### 2.5. Quantification and Method Validation

Solvent blanks, process blanks, and independent test standards (50 ng mL^−1^ standard solution) were run in sequence to check for residue, background contamination, and system performance. The reported quantitative values of each analyte in the samples were required to have the same retention time as its calibration standard and the same ion ratio. An independent test standard was inserted approximately every 10 injections to verify the stability of the instrument. If the concentration computed and the retention time of the instrument exceeded the standard value, the instrument was rebalanced and the sample quantified again to ensure the accuracy of the data results. All data processing was performed using Analyst Software Version 1.6.2. Limits of detection and limits of quantification of all analytes were calculated with signal/noise ratios of 3 and 10, respectively. The signal/noise ratios were obtained by averaging the results of three samples with the lowest spiked concentration [40]. The estimated limits of quantification values were used as the sample addition concentrations for seven environmental matrix spiked extracts to obtain the method detection limits (MDLs) and method quantitative limits (MQLs) [38]. The MDL of a target analyte was calculated according to the following equation:

(1) $$\begin{array}{c} \text{MDL}=t_{\left(n-1,1-\alpha =0.99\right)}\times \left(\text{SD}\right), \end{array}$$

where *t*_(*n* − 1,1 − *α*=0.99)_ and SD are, respectively, the t-distribution with a degree of freedom of n-1 and a confidence level of 99% and the standard deviation of the results of measurements.

In addition, matrix effects were estimated by the analyte concentration differences in environmental substrate sample extracts and solvents both spiked with 10 ng mL^−1^ of all analytes [37], i.e.,

(2) $$\begin{array}{c} \text{Matrix effects }\left(\%\right)=\frac{S_{\text{matrix}}}{S_{\text{standard}}}\times 100\%, \end{array}$$

where *S*_matrix_ and *S*_standard_ are chromatographic peak areas of matrix blank and standard solution, respectively. The matrix effects values of less or greater than 100% represent signal suppression or enhancement, respectively (Supplemental Data; Table S3).

## 3. Results and Discussion

### 3.1. Optimization of LC-MS/MS Conditions

The separation of 47 target analytes was optimized with an Agilent Eclipse Plus C_18_ column (100 mm × 2.1 mm, 1.8 *μ*m), a Sigma-Aldrich C_18_ column (100 mm × 4.6 mm, 3 *μ*m), and a Thermo Betasil C_18_ column (100 mm × 2.1 mm, 5 *μ*m) with the mobile phase of MilliQ water containing 0.1% formic (mobile phase A) and acetonitrile (mobile phase B). When the flow rates of the 1.8 *μ*m, 3 *μ*m, and 5 *μ*m columns were 0.25, 0.5, and 0.5 mL min^−1^, respectively, the analytes were well resolved and had sharp peak shapes. In terms of separation efficiency, the separation of 1.8 *μ*m and 3 *μ*m columns was better than that of 5 *μ*m column. The three isomers (sulfameter, sulfamethoxypyridazine, and sulfamonomethoxine) were not well separated under 5 *μ*m column conditions, and some of the analytes have a large half-width. Considering the chromatographic resolution and analysis time as well as reducing the high-pressure injection protection instrument, a comprehensive comparison of the final selection was a 3 *μ*m column.

**Figure 1:**
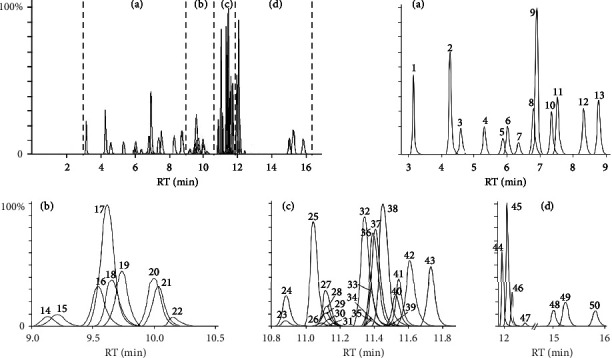
Typical reconstructed MRM chromatograms for the analytes: 50 compounds acquired under positive mode. The peak number of 1 to 50 correspond to pymetrozine, omethoate, lincomycin, dinotefuran, thiamethoxam, nitenpyram, sulfacetamide, sulfadiazine, monocrotophos, sulfathiazole, sulfapyridine, sulfamerazine, parathion, flonicamid, spiramycin, sulfamethazine-*d*_4_, sulfamethazine, sulfamethoxypyridazine-*d*_3_, sulfameter, sulfamethizole, sulfamethoxypyridazine, clindamycin, tilmicosin-*d*_3_, tilmicosin, oleandomycin, imidacloprid-*d*_4_, sulfamonomethoxine, imidacloprid, clothianidin-*d*_3_, clothianidin, erythromycin, dimethoate, sulfachloropyridazine, parathion-methyl, imidaclothiz, acetamiprid-*d*_3_, acetamiprid, sulfadoxine, leucomycins, sulfamethoxazole-*d*_4,_ sulfamethoxazole, josamycin, sulfisoxazole, thiacloprid, sulfadimethoxine, sulfaphenazole, dichlorvos, testosterone, methidathion, and methyltestosterone.

**Figure 2:**
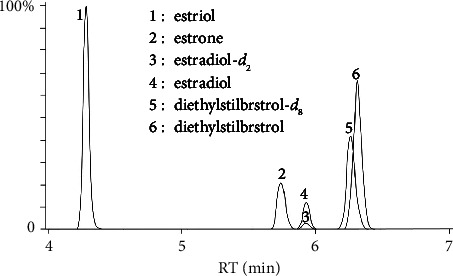
Typical reconstructed MRM chromatograms for the analytes: 6 compounds acquired under negative mode. The peak numbers from 1 to 6 correspond to estriol, estrone, estradiol-*d*_2_, estradiol, diethylstilbrstrol-*d*_8_, and diethylstilbrstrol.

The addition of additives to the mobile phase could improve the peak shapes of the analytes and their responses on the instrument [39]. Some studies added formic acid and ammonium acetate to the mobile phase to increase the response of the antibiotics on the instrument, and the organic phase was selected from both methanol and acetonitrile [38, 39]. We compared the difference between methanol and acetonitrile in the mobile phase. In general, the system pressure of methanol was higher than that of acetonitrile. Although acetonitrile is not recommended as a toxic solvent, the higher system pressure was not conducive to the long-term stable operation of the column and instrument. It is reasonable to choose acetonitrile as the mobile phase. We also compared the effects of the proportion of additive formic acid (0.1%, 0.2%, and 0.5% in volume) on the analytes in the mobile phase A. The addition of formic acid has improved the peak shape of the analytes, but the increase in the ratio of formic acid does not significantly enhance the response of the analytes on the instrument. In contrast, we found that by adding 0.1% ammonia to mobile phase A in negative ion mode, the response of the analytes on the instrument significantly improved. In order to reduce the switching time of the positive and negative ion modes and the use of the mobile phase, a 5 *μ*m column as the separation column for the negative ion mode was chosen. The optimized total elution time of the analytes is 17 and 8 min in positive and negative ion modes, respectively (Figures 1 and 2).

### 3.2. Optimization of Pretreatment Method Conditions

**Figure 3:**
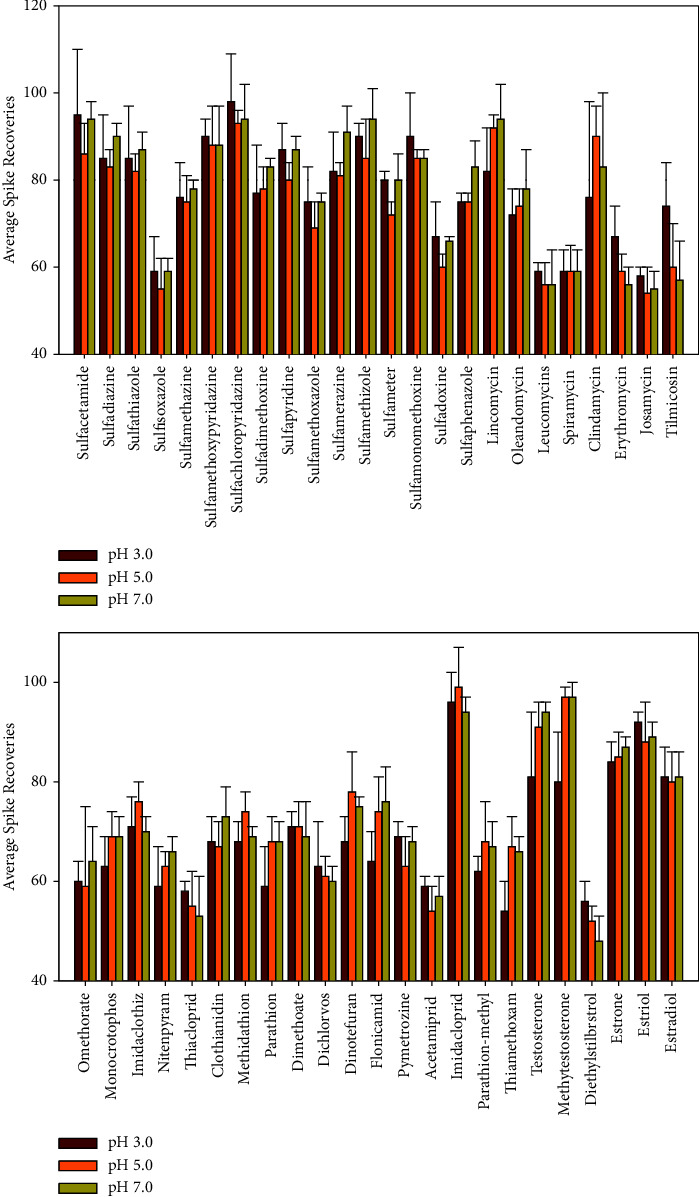
Extraction efficiencies of the solid-phase extraction column with Cleanert PEP-2 under different pH values (*n* = 3).

Studies have indicated that the addition of Na_2_EDTA to aqueous samples has increased the recoveries of MLs [43]. These studies [38, 39] also compared the effects of the quality of the addition of Na_2_EDTA on antibiotics. Na_2_EDTA (0.2 g) was added to promote the recovery of antibiotics. Extraction of all analytes in water samples was optimized by testing with the spiked samples at pH (3.0, 5.0, and 7.0). It showed that the recoveries of tilmicosin and erythromycin at pH 3.0 were higher than at other pH conditions, but lincomycin, clindamycin, testosterone, and methytestosterone at pH 7.0 were higher than at pH 3.0 (Figure 3). Shao et al. also verified that the extraction efficiency of solid-phase extraction cartridges with different pH values (3.0, 5.0, 7.0, and 9.0) indicated that the extraction efficiency of sulfonamides was not affected by pH [38]. But the extraction efficiency of tilmicosin and erythromycin was significantly better than other conditions at pH 3.0, which was similar to the optimization results in this study. The pH had slight effects on the spiked recoveries of OPs and NNIs. Although the spiked recoveries of some of the analytes were inhibited, in order to consider the low spiked recoveries of MLs and ensure acceptable recoveries of other analytes, a pH of 3.0 was finally selected. Therefore, conditions (0.2 g Na_2_EDTA and pH 3.0) were finally selected as they provided acceptable recoveries from the spiked samples.

### 3.3. Method Performance Validation

Method performance was assessed in terms of calibration linearity and range, recovery efficacy, matrix interferences, and method detection limits. Accurate quantification was performed using a seven-point calibration curve and showed a good linear relationship for analytes in the range of 0.1 to 100 ng mL^−1^. The linearity of all calibration curves was *S* > 0.99. The right linear range was selected for accurate quantification of environmental samples. If the quantitative concentration of a sample exceeded the linear range, a diluted sample was reinjected for accurate quantitation.

The recoveries of analytes were between 41% and 127%, indicating that the separation and purification of the samples met the requirements. The loss of analytes in the solid-phase extraction process is mainly due to the key factors such as the activation of the filler, the amount of adsorption, and the control of the loading flow rates. Quantitative use of the internal standard method minimizes the negative effects of matrix effects. Experimental analysis of water samples in the eight major runoff outlets of the PRD showed that some of the target analytes were detected, and the recoveries (average ± standard deviation) of sulfamethazine-*d*_4_, sulfamethoxypyridazine-*d*_3_, acetamiprid-*d*_3_, clothianidin-*d*_3_, and diethylstilbrstrol-*d*_8_ were 68 ± 7%, 79 ± 11%, 73 ± 5%, 95 ± 11%, and 71 ± 10%. The relative standard deviation was equal to or less than 20% except for individual analytes.

The matrix effects in LC-MS/MS are caused by the ionisation efficiency of the electrospray interface affected by the coeluting component of the analytes, which are manifested by ion enhancement or inhibition. It is usually corrected by the internal standard method or matrix standard solution to compensate for matrix effects. The matrix effects of most target analytes were in the range of 27–151%. The matrix effects were compensated by adding four suitable internal standards to the matrix. The matrix effects are effectively compensated by matrix-matched calibration curves and isotope dilution techniques. Due to the complexity and variability of the samples, it is not appropriate to have a matrix-matched calibration curve that takes into account economic issues.

MDLs are closely related to instrument sensitivity, concentration factor, and injection volume. This method has a concentration factor of 1000 and an injection volume of 3 *μ*L with a Triple Quadrupole 5500 electrospray ionization-tandem mass spectrometer. We have calculated the MQLs of all analytes by (1). MQLs of the SAs, MLs, HMs, OPs, and NNIs were in the range of 0.53–1.49 ng L^−1^, 0.40–5.49 ng L^−1^, 0.40–3.16 ng L^−1^, 0.54–1.98 ng L^−1^, and 0.50–2.07 ng L^−1^.

### 3.4. Application of Environmental Samples

This method was used to analyze the environmental water samples collected at eight major runoff outlets of the PRD, South China, on August 11, 2018. The concentrations of target analytes in the environmental samples are presented in Table 4. The five sulfonamides detected in the eight major runoff outlets are also found in most rivers in China. And erythromycin is also present in many large rivers in China, but lincomycin and clindamycin, which have lower detection rates in most rivers, were detected in water samples. The concentration of parathion was as high as 154 ng L^−1^. The use of parathion was banned in 2003, but high levels of parathion were still detected in the water samples, indicating a high background in the environment and slow release through surface runoff and rain washout. The three neonicotinoids (acetamiprid, clothianidin, and imidacloprid), which are used in large amounts, were also presented in the water sample. It can be seen that the concentration of NNIs was generally higher than that of OPs, which may restrict the use of OPs and the replacement of NNIs as OPs in agricultural production. The average recoveries of the method were 68–95%, indicating that the method is simple and effective for actual environmental waters. This optimization method has been successfully applied to the analysis of the waters at eight major runoff outlets in the PRD. The frequent detection of various human veterinary drugs and pesticide residues indicated that the PRD has received pollution, and there may be certain ecological risks that should be given sufficient attention. A simple, economical, and powerful tool has been provided for other environmental waters.

## Figures and Tables

**Table 1:** Instrument conditions for human and veterinary drugs and pesticide residues analysis.

Ionization modes		ESI+							ESI−					
LC condition	Mobile phase	A	0.1% formic acid in water						A	0.1% ammonia in water				
		B	Acetonitrile						B	Acetonitrile				
	Gradient list	Time (min)	0	8	10	14	14.1	17	Time (min)	0	3	7	7.1	8
		A (%)	90	75	40	40	90	90	A (%)	90	10	10	90	90
		B (%)	10	25	60	60	10	10	B (%)	10	90	90	10	10
	Total flow (mL min^−1^)	0.5							0.5					
	Column	Sigma-Aldrich C_18_ column							Thermo Betasil C_18_ column					
		(100 mm × 4.6 mm, 3 *μ*m)							(100 mm × 2.1 mm, 5 *μ*m)					
	Column temperature (°C)	40							40					

MS condition	Ion source gas1 (psi)	40							40					
	Ion source gas2 (psi)	40							40					
	Curtain gas (psi)	30							30					
	Collision gas (psi)	7							7					
	Temperature (°C)	450							450					
	Ionspray voltage (V)	5000							−4500					

**Table 2:** LC-MS/MS acquisition parameters for analytes in positive mode.

Analytes	M.W.	R.T.^a^ (min)	Q1	Q3	DP^b^	CE^c^	EP^d^	CXP^e^	Dwell^f^	Peak no.
Sulfacetamide	214.24	6.34	215.1	156/92	50	15/32	10	10	10	7
Sulfapyridine	249.29	7.52	250.1	156/108	33	22/32	10	14	10	11
Sulfadiazine	250.28	6.8	251.1	156/108	26	21/32	14	10	10	8
Sulfamethoxazole	253.28	11.55	254.1	156/92	57	22/35	6	10	5	41
Sulfathiazole	255.31	7.36	256.1	156/108	25	20/30	6	14	10	10
Sulfamerazine	264.3	8.32	265.1	156/108	24	23/33	8	12	10	12
Sulfisoxazole	267.3	11.73	268.1	156/108	10	19/31	6	10	5	43
Sulamethizole	270.33	10	271.1	156/108	30	20/31	14	16	10	20
Sulfamethazine	278.33	9.61	279.1	156/108	30	23/34	14	10	10	17
Sulfameter	280.31	9.73	281.1	156/126	30	23/31	12	10	10	19
Sulfamethoxypyridazine	280.3	10.03	281.1	156/108	30	33/30	12	12	10	21
Sulfamonomethoxine	280.3	11.12	281.1	156/108	30	39/28	10	10	10	27
Sulfachloropyridazine	284.72	11.38	285.1	156/108	20	20/33	12	14	10	33
Sulfadoxine	310.33	11.46	311.1	156/108	35	25/28	12	12	10	38
Sulfadimethoxine	310.33	12.06	311.1	156/92	40	33/38	10	10	10	45
Sulfaphenazole	314.36	12.15	315.1	156/108	41	27/34	10	8	10	46
Lincomycin	406.54	4.57	407.1	126.1/359.2	33	33/25	12	10	10	3
Clindamycin	424.98	10.22	426.2	126.1/378.2	39	33/27	6	18	10	22
Oleandomycin	687.86	11.05	688.5	158.1/544.4	48	20/32	14	8	10	25
Erythromycin	733.93	11.16	734.4	158.1/576.4	52	36/32	6	18	5	31
Leucomycins	785.96	11.51	786.3	174.1/229.1	27	39/39	10	14	10	39
Josamycin	827.99	11.61	828.5	174.1/229.1	33	43/39	10	14	10	42
Spiramycin	843.06	9.21	844.3	174.2/616.3	50	39/30	6	8	10	15
Tilmicosin	869.13	10.89	869.6	696.5/174.1	13	57/54	12	14	5	24
Testosterone	288.42	15.02	289.2	109/253.2	51	31/24	14	10	10	48
Methyltestosterone	302.45	15.81	303.1	97/109	63	33/35	8	8	10	50
Dichlorvos	220.98	12.42	221.2	109/127.4	75	17/25	12	14	10	47
Monocrotophos	223.16	6.91	224.1	127/193	76	5/12	12	18	10	9
Omethoate	213.19	4.26	214	183/155	90	12/20	10	18	10	2
Methidathion	302.33	15.24	303.1	145.4/85.5	110	10/20	12	14	10	49
Parathion-methyl	263.21	11.38	264.2	180.9/231.8	100	21/19	8	8	10	34
Parathion	291.2	8.77	292.2	211/235.9	20	16/20	16	16	10	13
Dimethoate	229.12	11.35	230	198.9/125	75	5/20	10	18	10	32
Dinotefuran	202.21	5.32	203.3	129.1/114	120	17/17	6	16	10	4
Pymetrozine	217.23	3.15	218.1	114/105.1	115	18/20	8	8	10	1
Imidaclothiz	261.45	11.38	262.1	179/122.2	60	23/35	14	14	10	35
Flonicamid	229.16	9.15	230.1	89.9/198.9	42	44/11	8	14	10	14
Nitenpyram	270.72	6.02	271.3	180.9/99.2	95	30/18	6	16	10	6
Thiacloprid	252.72	11.97	253	131.9/126	100	18/18	10	16	10	44
Acetamiprid	222.67	11.41	223.3	78.2/126	100	48/25	6	14	10	37
Clothianidin	249.68	11.13	250.1	124.8/169.1	118	27/12	8	16	10	30
Imidacloprid	255.66	11.12	256	186/209.1	90	15/20	6	16	10	28
Thiamethoxam	291.71	5.95	293	122.9/211.2	45	15/15	6	16	10	5
Sulfamethoxazole-*d*_4_	257.28	11.53	258.1	160/95.9	76	21/34	8	16	10	40
Sulfamethazine-*d*_4_	282.33	9.54	283.1	186/124.1	40	23/32	10	10	10	16
Sulfamethoxypyridazine-*d*_3_	283.3	9.64	284.1	155.9/108	67	23/32	6	14	10	18
Tilmicosin-*d*_3_	872.13	10.88	872.7	696.5/177.1	17	56/55	10	16	10	23
Acetamiprid-*d*_3_	225.67	11.4	226.2	98.9/125.9	79	48/28	10	16	10	36
Clothianidin-*d*_3_	252.68	11.12	253.1	132.1/172	77	24/17	6	16	10	29
Imidacloprid-*d*_4_	259.66	11.09	260.1	175.2/213	74	12/19	6	12	10	26

**Table 3:** LC-MS/MS acquisition parameters for analytes in negative mode.

Analytes	M.W.	R.T.^a^ (min)	Q1	Q3	DP^b^	CE^c^	EP^d^	CXP^e^	Dwell^f^	Peak no.
Diethylstilbrstrol	268.35	6.31	267.3	237.1/251.1	−100	−37/-32	−10	−12	50	6
Estradiol	272.38	5.93	271.1	144.8/183.1	−84	−53/-53	−8	−10	50	4
Estrone	270.37	5.74	269.1	145/183.2	−83	−48/-45	−10	−10	50	2
Estriol	288.38	4.28	287.1	145.1/171	−85	−48/-55	−8	−12	50	1
Diethylstilbrstrol-*d*_8_	276.35	6.26	275.2	213/259.1	−50	−49/-36	−10	−12	10	5
Estradiol-*d*_2_	274.38	5.91	273.1	147/185	−89	−51/-52	−8	−10	10	3

The first MRM transitions were used for quantification. ^a^Retention time. ^b^Declustering potential. ^c^Collision energy. ^d^Entrance potential. ^e^Collision cell exit potential. ^f^Dwell time.

**Table 4:** Concentrations of analytes in water at eight major runoff outlets in the PRD, South China.

Analytes (ng/L)	HE	YM	MD	JT	HT	HQ	JM	HM
Sulfadiazine	7.9	5.3	11.0	7.4	10.2	6.9	6.8	4.3
Sulfamethoxazole	9.9	5.9	10.8	12.0	9.2	10.8	10.4	11.1
Sulfamethazine	3.2	6.9	7.6	7.1	4.5	8.1	7.3	20.9
Sulfamonomethoxine	5.7	8.9	9.0	8.2	7.1	8.6	7.7	10.2
Sulfachloropyridazine	3.4	3.1	3.9	4.3	5.2	4.5	4.0	3.5
Lincomycin	2.6	4.4	1.8	2.3	2.4	2.1	3.6	8.6
Clindamycin	1.6	1.6	1.5	0.8	1.1	1.3	1.4	4.7
Erythromycin	3.1	5.5	4.2	1.4	2.7	2.9	4.5	4.0
Omethorate	<MQL^a^	2.1	<MQL	0.9	0.6	<MQL	0.6	1.3
Dimethoate	3.6	2.7	2.0	3.9	3.4	3.1	3.1	5.5
Acetamiprid	6.4	6.7	6.4	5.2	5.2	6.7	7.6	15.0
Clothianidin	41	23	38	32	26	40	34	15
Imidacloprid	44	29	40	40	26	45	42	28
Methidathion	1.4	3.3	<MQL	0.7	<MQL	0.7	0.9	1.3
Parathion	154	101	132	126	83	139	131	41

The eight major runoff outlets are labeled as humen (HM), jiaomen (JM), honqimen (HQ), hengmen (HE), modaomen (MD), jitimen (JT), hutiaomen (HT), and yamen (YM). ^a^Below method quantitation limit. All other analytes are below the method quantitation limit.

## Data Availability

The data used to support the findings of this study are included within the supplementary information file.

## Supplementary Materials

Supplementary Materials Table S1: analyte details. Table S2: geographic information of sampling sites and sampling time. Table S3: recoveries (*n* = 3), method detection limits, and method quantitation limits of human and veterinary drugs and pesticide residues from surface water.
