# Supplementary material for: Liquid Chromatography-Tandem Mass Spectrometry Detection of Human and Veterinary Drugs and Pesticides in Surface Water
Source: Int J Anal Chem. 2023 Oct 16;2023:6350669. doi: 10.1155/2023/6350669 (PMC10593548; doi:10.1155/2023/6350669)
Supplement: Supplementary Materials — Table S1: analyte details. Table S2: geographic information of sampling sites and sampling time. Table S3: recoveries (n = 3), method detection limits, and method quantitation limits of human and veterinary drugs and pesticide residues from surface water. [file 6350669.f1.docx]

**Table S1. Details of the source of purchase of all analytes.**

Analytes Source of the standards analytes Source of the standards

Sulfonamides Sulfacetamide O2si Smart Solutions Macrolides Lincomycin Chem Tek

Sulfapyridine (Charleston, SC, USA) Clindamycin (Worcester, MA, USA)

Suladiazine Oleandomycin

Sulfamethoxazole Erythromycin

Sulfathiazole Leucomycins

Sulfamerazine Josamycin

Sulfisoxazole Spiramycin

Sulfamethizole Tilmicosin

Sulfamethazine Hormones Testosterone

Sulfameter Methytestosterone

Sulfamethoxypyridazine Diethylstilbrstrol

Sulfamonomethoxine Estrone

Sulfachloropyridazine Estradiol

Sulfadimethoxine Estriol

Sulfadimethoxine Neonicotinoids Pymetrozine Manhage Bio-Tech

Sulfaphenazole Flonicamid (Beijing, China)

Organophosphorus Dichlorvos O2si Smart Solutions Nitenpyram

Monocrotophos (Charleston, SC, USA) Thiacloprid

Omethoate Acetamiprid

Methidathion Clothianidin

Parathion-methyl Imidacloprid

Parathion Thiamethoxam

Dimethoate Dinotefuran

Imidaclothiz

**Table S2**. **Geographic information of sampling sites and sampling time.**

Sites^a^ Cross section Latitude Longitude Sampling Time

(N) (E) （Aug. 11）

HM Dahu 22°48.18′ 113°35.04′ 18:30

JM Nansha 22°44.64′ 113°33.84′ 19:10

HQ Wanqingsha 22°42.00′ 113°29.70′ 17:57

HE Hengmen 22°34.44′ 113°31.14′ 18:32

MD Denglongshan 22°13.74′ 113°23.82′ 18:20

JT Huangjin 22°8.16′ 113°17.16′ 18:25

HT Xipaotai 22°13.32′ 113°7.32′ 18:10

YM Huangyong 22°16.98′ 113°4.5′ 18:00

^a^ The eight runoff outlets are labeled as Humen (HM), Jiaomen (JM), Honqimen (HQ), Hengmen (HE), Modaomen (MD), Jitimen (JT), Hutiaomen (HT), and Yamen (YM).

**Table S3. Recoveries (n=3), method detection limits (MDLs) and method quantitation litmits (MQLs) of human and veterinary drugs and pesticide residues from surface water.**

analytes IS^a^ Surface water (ng/L)

10 50 100 Matrix (%)^c^ Matrix_IS_(%)^d^ MDLs MQLs

Sulfacetamide Sulfamethoxazole-*d*4 127±9^b^  99±11 98±7 110±2 103±5 0.42 1.25

Sulfapyridine Sulfamethoxazole-*d*4 119±4 104±9 93±6 104±2 95±6 0.26 0.77

Suladiazine Sulfamethoxazole-*d*4 125±9 107±6 96±8 109±7 101±4 0.50 1.49

Sulfamethoxazole Sulfamethoxazole-*d*4 56±2 60±12 70±8 87±9 94±5 0.41 1.23

Sulfathiazole Sulfamethoxazole-*d*4 104±4 101±4 88±6 108±6 103±4 0.37 1.10

Sulfamerazine Sulfamethoxazole-*d*4 120±6 106±5 94±7 112±6 102±4 0.43 1.30

Sulfisoxazole Sulfamethoxazole-d4 49±1 50±6 54±5 83±9 88±4 0.28 0.85

Sulfamethizole Sulfamethoxazole-d4 100±3 94±3 86±10 110±7 96±5 0.49 1.46

Sulfamethazine Sulfamethoxazole-d4 97±5 90±12 88±8 112±7 98±3 0.22 0.67

Sulfameter Sulfamethoxazole-d4 97±3 87±8 89±8 109±6 103±3 0.22 0.65

Sulfamethoxypyridazine Sulfamethoxazole-d4 114±4 103±5 88±10 110±9 96±5 0.18 0.53

Sulfamonomethoxine Sulfamethoxazole-d4 126±3 115±16 108±10 151±15 110±4 0.47 1.42

Sulfachloropyridazine Sulfamethoxazole-d4 75±3 78±11 81±8 133±4 104±5 0.27 0.80

Sulfadoxine Sulfamethoxazole-d4 52±3 71±11 81±7 88±5 92±4 0.31 0.94

Sulfadimethoxine Sulfamethoxazole-d4 65±1 64±4 66±7 99±7 96±4 0.27 0.81

analytes IS^a^ Surface water (ng/L)

10 50 100 Matrix (%)^c^ Matrix_IS_(%)^d^ MDLs MQLs

Sulfaphenazole Sulfamethoxazole-*d*4 65±2 70±9 74±3 113±4 104±3 0.30 0.89

Lincomycin Tilmicosin-*d*_3_ 61±7 66±21 67±16 107±4 101±5 0.34 1.01

Clindamycin Tilmicosin-*d*_3_ 59±13 66±12 62±7 105±1 95±3 0.29 0.86

Oleandomycin Tilmicosin-*d*_3_ 51±6 69±3 74±5 96±5 102±3 0.41 1.23

Erythromycin Tilmicosin-*d*_3_ 71±14 64±5 63±4 92±11 95±5 0.62 1.87

Leucomycins Tilmicosin-*d*_3_ 43±7 47±5 49±4 39±11 86±4 0.41 1.23

Josamycin Tilmicosin-*d*_3_ 41±10 47±9 46±6 74±10 89±5 0.25 0.76

Spiramycin Tilmicosin-*d*_3_ 56±4 61±12 58±7 27±13 87±5 1.83 5.49

Tilmicosin Tilmicosin-*d*_3_ 42±12 42±11 46±14 122±4 103±3 0.13 0.40

Dichlorvos Imidacloprid-*d*_4_ 60±1 53±6 44±4 91±9 95±4 0.61 1.83

Monocrotophos Imidacloprid-*d*_4_ 87±4 79±8 73±4 99±4 103±5 0.39 1.18

Omethoate Imidacloprid-*d*_4_ 67±5 71±11 76±9 87±6 86±3 0.20 0.59

Methidathion Imidacloprid-*d*_4_ 63±2 65±8 63±2 96±4 103±2 0.18 0.54

Parathion-methyl Imidacloprid-*d*_4_ 64±1 82±12 80±5 108±4 101±5 0.66 1.98

Parathion Imidacloprid-*d*_4_ 98±7 87±14 80±2 117±9 104±6 0.23 0.70

Dimethoate Imidacloprid-*d*_4_ 62±4 67±6 73±7 53±5 89±4 0.35 1.05

Dinotefuran Imidacloprid-*d*_4_ 66±3 88±4 93±5 106±2 97±3 0.65 1.94

analytes IS^a^ Surface water (ng/L)

10 50 100 Matrix (%)^c^ Matrix_IS_(%)^d^ MDLs MQLs

Pymetrozine Imidacloprid-*d*_4_ 101±17 86±5 85±10 87±5 96±4 0.48 1.43

Imidaclothiz Imidacloprid-*d*_4_ 72±1 72±4 84±12 108±2 98±3 0.17 0.52

Flonicamid Imidacloprid-*d*_4_ 46±4 52±5 57±9 70±3 86±5 0.38 1.14

Nitenpyram Imidacloprid-*d*_4_ 83±9 73±10 66±2 95±2 96±4 0.69 2.07

Thiacloprid Imidacloprid-*d*_4_ 43±2 47±5 52±7 83±3 86±4 0.17 0.50

Acetamiprid Imidacloprid-*d*_4_ 42±3 47±7 56±7 65±3 85±4 0.27 0.82

Clothianidin Imidacloprid-*d*_4_ 66±1 69±6 66±4 98±9 94±5 0.27 0.81

Imidacloprid Imidacloprid-*d*_4_ 90±1 101±6 89±3 114±9 96±7 0.29 0.88

Thiamethoxam Imidacloprid-*d*_4_ 89±8 90±16 75±3 108±2 89±6 0.68 2.05

Testosterone Tilmicosin-*d*_3_ 84±5 88±2 80±4 100±5 93±4 0.15 0.44

Methytestosterone Tilmicosin-*d*_3_ 87±7 85±2 85±2 104±9 89±7 0.13 0.40

Diethylstilbrstrol Estradiol-*d*_2_ 52±6 51±6 49±2 101±4 96±3 0.29 0.88

Estrone Estradiol-*d*_2_ 83±9 77±6 71±1 101±4 87±2 1.05 3.16

Estradiol Estradiol-*d*_2_ 87±1 83±2 86±4 100±3 89±4 0.53 1.58

Estriol Estradiol-*d*_2_ 66±9 68±13 69±6 107±2 102±4 0.83 2.49

^a^ Internal standards.

^b^ Mean(%)±standard deviation(%).

^c^ <100% means matrix suppression effect, >100% means matrix enhancement effect.

^d^ Matrix effect after adding internal standards.
